# Salidroside, A Natural Antioxidant, Improves β-Cell Survival and Function via Activating AMPK Pathway

**DOI:** 10.3389/fphar.2017.00749

**Published:** 2017-10-18

**Authors:** Linjie Ju, Xiaohua Wen, Chunjun Wang, Yingjie Wei, Yunru Peng, Yongfang Ding, Liang Feng, Luan Shu

**Affiliations:** ^1^Affiliated Hospital of Integrated Traditional Chinese and Western Medicine, Nanjing University of Chinese Medicine, Nanjing, China; ^2^Key Laboratory of New Drug Delivery System of Chinese Materia Medica, Jiangsu Provincial Academy of Chinese Medicine, Nanjing, China

**Keywords:** type 2 diabetes, β-cells, salidroside, oxidative stress, AMPK

## Abstract

**Aim:** The enhanced oxidative stress contributes to progression of type 2 diabetes mellitus (T2DM) and induces β-cell failure. Salidroside is a natural antioxidant extracted from medicinal food plant *Rhodiola rosea*. This study was aimed to evaluate protective effects of salidroside on β-cells against diabetes associated oxidative stress.

**Methods and Results:** In diabetic db/db and high-fat diet-induced mice, we found salidroside ameliorated hyperglycemia and relieved oxidative stress. More importantly, salidroside increased β-cell mass and β-cell replication of diabetic mice. Mechanism study in Min6 cells revealed that, under diabetic stimuli, salidroside suppressed reactive oxygen species production and restore mitochondrial membrane potential (ΔΨm) via reducing NOX2 expression and inhibiting JNK–caspase 3 apoptotic cascade subsequently to protect β-cell survival. Simultaneously, diabetes associated oxidative stress also activated FOXO1 and triggered nuclear exclusion of PDX1 which resulted in β-cell dysfunction. This deleterious result was reversed by salidroside by activating AMPK-AKT to inhibit FOXO1 and recover PDX1 nuclear localization. The efficacy of salidroside in improving β-cell survival and function was further confirmed in isolated cultured mouse islets. Moreover, the protective effects of salidroside on β-cells against diabetic stimuli can be abolished by an AMPK inhibitor compound C, which indicated functions of salidroside on β-cells were AMPK activation dependent.

**Conclusion:** These results confirmed beneficial metabolic effects of salidroside and identified a novel role for salidroside in preventing β-cell failure via AMPK activation. Our finding highlights the potential value of *Rhodiola rosea* as a dietary supplement for diabetes control.

## Introduction

Oxidative stress is a hallmark in the development of many diseases such as cancer, Parkinson’s disease, Alzheimer’s disease, and diabetes (Valko et al., 2007). Reactive oxygen species (ROS) is one of the most important factors that result in insulin resistance and β-cell dysfunction (Supale et al., 2012; Gerber and Rutter, 2017). NADPH oxidase 2 (NOX2) is a major enzyme to modulate ROS generation in β-cells (Li et al., 2012). Evidence shows that mitogen-activated protein kinase (MAPK) pathways are activated in the presence of intracellular ROS to trigger β-cell apoptosis (Sidarala and Kowluru, 2017). AMPK/mTOR signaling mediates protecting β-cells against diabetic oxidative stress (Wang et al., 2016). Oxidative stress also induces activation of FOXO1 which suppresses the function of PDX1 to reduce insulin expression consequently (Ding et al., 2014).

*Rhodiola rosea* (Golden Root) is a high-value functional and medicinal food plant, commonly used for healthcare in China and other Asian countries, also registered in United Kingdom and throughout Europe as a commercially dietary supplement for the treatment of stress-induced fatigue, exhaustion, etc. (Booker et al., 2016). Salidroside, a bioactive constituent isolated from this plant, has potent antioxidant, anti-viral, neuroprotective, and hepatoprotective effects (Yu et al., 2007). For example, salidroside improved the impaired hippocampal neurogenesis in the rat model of Alzheimer’s disease through protecting NSCs by scavenging intracellular ROS (Qu et al., 2012).

So far, there are only few reports about hypoglycemic effects of salidroside which were focused on improving insulin sensitivity. For example, salidroside has been shown to stimulate glucose uptake in skeletal muscle cells by activating AMP-activated protein kinase (AMPK) (Li et al., 2008). A recent study reported that salidroside ameliorated insulin resistance through activation of a AMPK/PI3K/AKT/GSK3β pathway (Zheng et al., 2015).

We noticed that the direct effect of salidroside on regulating β-cell survival still remains unknown. β-cell failure is a common feature of most forms of diabetes. To prevent β-cell loss and to promote new β-cell formation would be a promising strategy for diabetes treatment. In the present study, we aimed to clarify whether salidroside could protect β-cell survival and function against diabetic conditions. The mechanism responsible for actions of salidroside will be investigated as well.

## Materials and Methods

### Reagent

Salidroside (purity > 98%) was purchased from the National Institute for the Control of Pharmaceutical and Biological Products (Beijing, China). Compound C was from Selleck (Houston, TX, United States).

### Animals and Experimental Design

All animals were housed in a temperature-controlled room with a 12-h/12-h light/dark cycle and were allowed free access to food and water during the course of experiments. Before the experiment, the mice were kept for 1 week to acclimatize them to the conditions. The 4-week-old male C57BL/6 mice (SLAC Laboratory Animals, Shanghai, China) were fed a high-fat diet (HFD) (p1400f, SLAC Laboratory Animals, Shanghai, China) (*n* = 16) or normal chow diet (*n* = 8). After 10 weeks of the HFD, salidroside intervention (100 mg/kg/day) was initiated by gavage once a day for 5 weeks. The control groups were given vehicle (saline). The 4-week-old male C57Bl/KsJ (BKS) mice (wild type, *n* = 8) and BKS.Cg-Dock7m +/+ Leprdb/J (db/db) mice (*n* = 16) were ordered from Model Animal Research Center of Nanjing University (Nanjing, China). Salidroside (100 mg/kg/day) was administrated orally by gavage once a day for 5 weeks. The control groups were given vehicle (saline). Fasting blood glucose and body weight of mice were monitored every 5 days. Glucose measurements were performed on blood drawn from the tail vein using a Glucometer (Accu-Chek Active; Roche Inc.). All animal experiments were conducted in accordance with Provisions and General Recommendation of Chinese Experimental Animals Administration Legislation and approved by the Research Animal Care Committee of Nanjing University of Chinese Medicine.

### Oral Glucose Tolerance Tests (OGTT)

After 5-week salidroside treatment, mice were fasted 12 h overnight for oral glucose tolerance test (OGTT) experiments. In the next morning, mice were taken glucose orally at a dose of 2 mg/g body weight. Then blood samples of mice were obtained at time points 0, 30, 60, 90, and 120 min for blood glucose measurements using a Glucometer (Accu-Chek Active; Roche Inc.).

### Analysis of Mouse Blood and Tissue Samples

Blood and tissue samples of mice were collected after 5-week salidroside treatment, and tissue samples were weighed. Serum insulin level was determined using a mouse insulin ELISA kit (RayBiotech, Norcross, GA, United States). Contents of total cholesterol (CHO), low-density lipoprotein cholesterol (LDL-C), high-density lipoprotein cholesterol (HDL-C), and triglycerol (TG) in serum were tested by commercial assay kits (Jiancheng Bioengineering Inc., Nanjing, China). Enzyme activities of Superoxide dismutase (SOD), Glutathione peroxidase (GPx), Catalase (CAT), and content of Malonaldehyde (MDA) were assessed using tissue lysate of adipose and pancreas by commercial assay kits (Jiancheng Bioengineering Inc., Nanjing, China). All measurements were performed and calculated according to the instructions of the assay kits.

### β-Cell Mass Measurement

Pancreas of mice were weighed, fixed with 4% paraformaldehyde and then embedded in paraffin to prepare 4 μm pancreatic sections. In brief, the paraffin sections of mouse pancreases were deparaffinized, rehydrated, antigen unmasking (Vector Laboratories, Inc., United States), blocking, and incubated overnight at 4°C with anti-insulin antibody (1:200 dilution) (ab7842, Abcam). In the next day, the sections were washed and stained by FITC secondary antibody. Then the β-cell areas and the entire tissue areas were scanned by a Nikon MEA53200 (Nikon, Japan) microscope. The cross-sectional areas of pancreas and β-cells were determined and calculated by NIS-Elements software (Nikon). The average β-cell mass of each mouse was calculated as follows: the islets/pancreas area ratio × the pancreatic weight. Five consecutive sections from each pancreas (6 mice per group) were used for β-cell mass measurements.

### Mouse Pancreatic Islets Isolation and Min6 Cell Culture

Mouse islets were isolated from 10-week-old male C57BL/6 mice or 10-week-old male dbdb mice by common bile duct perfusion using Collagenase type 4 (Sunshine Biotechnology, China). Infuse 2 ml of collagenase solution (2 mg/ml) into a mouse pancreas. Transfer infused pancreas to 2 ml collagenase solution in 50-ml falcon tube and digest at 37°C for 20–25 min in water bath. Shake tubes every 5 min. Stop the reaction by adding 30 ml ice-cold quenching buffer and shake, then centrifuge at 1200 rpm for 3 min at 4°C. Remove the supernatant, and repeat wash step by quenching buffer twice. Resuspend pellets with 13 ml of cold Histopaque-1077 (Sigma) by pipetting. Fill to 25 ml with cold serum (-) RPMI media carefully with transfer pipettes. Add media slowly and gently to make clear 2 layers. Centrifuge 2500 rpm, 24 min, 4°C (Slow acceleration, no brake). Pick up floating islets in the interface between media and Histopaque with transfer pipettes, then wash islets twice using full media. Isolated islets were transferred to 6-well plates (20–30 islets/well) and cultured in RPMI 1640 containing 11.1 mmol/l glucose, 100 U/ml penicillin, 100 mg/ml streptomycin, and 10% FBS (All from Gibco). The Min6 cell line was a kind of gift from Prof. Dongming Su (Nanjing Medical University, China) and maintained in 5 mM glucose DMEM, supplemented with 10% FBS, 50 μmol/L b-mercaptoethanol (Sigma), 100 U/ml penicillin, and 0.1 mg/ml streptomycin in 5% CO_2_ at 37°C.

### Immunofluorescence Staining

Cultured mouse islets in 6-well plates were fixed with 4% paraformaldehyde followed by permeabilization with 0.5% Triton X-100. The 4-μm paraffin sections of mouse pancreas were deparaffinized, rehydrated, and antigen unmasking. Then islets or sections were incubated with blocking buffer for 1 h at RT followed by incubating overnight at 4°C with antibody of insulin (Abcam), or Ki67 (1:50) (BD Pharmingen), or PDX1 (1:100) (Abcam). In the next day, islets or sections were washed by TBS and incubated by FITC- or Cy3-conjugated secondary antibodies (1:400) (Abcam) for 1 h at RT. β-cell apoptosis of pancreatic setions was detected by the commercial TUNEL staining kit according to the manufacturer’s instructions (TUNEL Brightred Apoptosis Detection Kit,Vazyme Biotech Co.). β-cell apoptosis of cultured islets was analyzed by the commercial TUNEL staining kit (*In Situ* Cell Death Detection Kit, TMR red; Roche Diagnostics). Slides were mounted with Vectashield Mounting Medium containing 4′6-diamidino-2-phenylindole (DAPI) as nuclear dye (Vector Laboratories, Inc., United States). Fluorescence was detected and analyzed using an Nikon MEA53200 (Nikon) microscope.

### Nuclear Fractionation

Nuclear and cytoplasm extractions of Min6 cells were performed according to the instructions of NE-PER Nuclear and Cytoplasm Extraction Reagents (Pierce biotechnology, United States). For each treatment, 1 × 10^6^ Min6 cells were used for nuclear fractionation. The purity of fractions was analyzed by probing the membranes with anti-GAPDH for cytosolic and anti-PARP for nuclear extracts.

### Glucose-Stimulated Insulin Secretion (GSIS)

For acute insulin release, cultured mouse islets (20–30 islets/well in 6-well plates) were washed by PBS twice and pre-incubated in 1 ml Krebs-Ringer bicarbonate buffer (KRB) containing 2.8 mM glucose for 30 min. The KRB was removed and replaced by 1 ml KRB containing 2.8 mM glucose for 1 h incubation (collected this 2.8KRB as basal). Then islets were incubated in KRB containing 16.7 mM glucose for 1 h (collected this 16.7KRB as stimulated). For insulin content, cells were extracted with 0.18 N HCl in 70% ethanol. Insulin contents were determined using a mouse insulin ELISA kit (RayBiotech, Norcross, GA, United States). The insulin stimulatory index of islets is calculated as stimulated/basal. Three wells per treatment group were used.

### Western Blot Analysis

Min6 cells were washed in PBS and lysed (Beyotime Biotech, Shanghai, China). For each treatment, 20 μg proteins were used for western blotting analysis. The following primary antibodies were used at 1:1000 dilution: p-JNK (#9255), JNK (#9258), Actin (#4967), c-Casp3 (#9661), p-FOXO1 (#9461), FOXO1 (#9462), p-AMPKα (#2535), AMPKα (#2532), p-AKT (#9271), PARP (#9542), GAPDH (#2118), (all from Cell Signaling); NOX2 (Abcam, #ab39072), PDX1 (Abcam, #ab47267) followed by incubation with horseradish-peroxidase-linked IgG peroxidase (1:5000, Vazyme Biotech, Nanjing, China). The bands were visualized and densities of the bands were analyzed using Tanon ChemImaging Systems (Tanon Science & Technology Co., Ltd., Shanghai, China).

### Measurement of ROS Production in Min6 Cells

To detect the ROS production in Min6 cells with different treatments, 1 × 10^6^ Min6 cells were collected, washed by PBS, and re-suspended in 500 μl serum (-) DMEM containing 10 μM DCFDA (Beyotime Biotech, China) for 30 min at 37°C in the dark. Then cells were washed 3 times with PBS and analyzed with flow cytometry (Becton Dickinson) with excitation set at 488 nm and emission at 530 nm.

### Measurement of Mitochondrial Membrane Potential (ΔΨm) in Min6 Cells

The ΔΨm changes in Min6 cells with different treatments were measured by uptake of rhodamine 123 (Rh123). The 1 × 10^6^ treated cells were harvested and washed twice with PBS, re-suspended in 500 μl serum (-) DMEM containing 2 μM Rh123 (Beyotime Biotech, China), and incubated at 37°C for 30 min in the dark. The samples were then immediately detected by flow cytometry (Becton Dickinson) with excitation set at 503 nm and emission at 527 nm.

### Statistical Analysis

Data are presented as means ± SD and were analyzed by paired, Student’s *t*-test or by analysis of variance with a Bonferroni correction for multiple group comparisons.

## Results

### Salidroside Alleviated Hyperglycemia in db/db and HFD Mice

It was reported that salidroside (100 mg/kg/day) showed strong glucose lowering effect on db/db mice which was similar to effect of metformin (200 mg/kg/day) (Zheng et al., 2015). For this reason, the dose of 100 mg/kg/day salidroside was used in our experiments.

**FIGURE 1:**
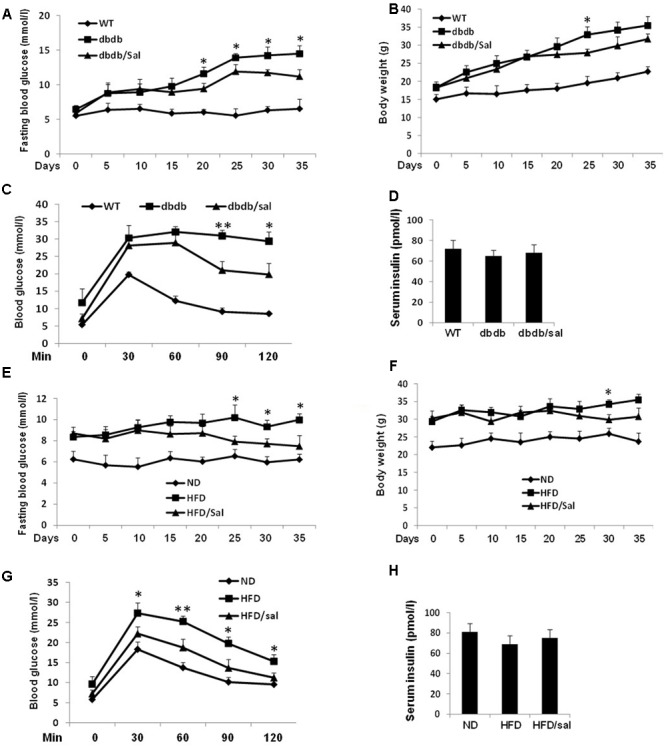
Salidroside improved glucose homeostasis in db/db and HFD mice. **(A,B)** Effects of salidroside (100 mg/kg/day) on the fasting glucose levels and body weights in db/db group mice. **(C)** After 5-week treatment, OGTT was performed in wild type (WT), salidroside, or vehicle-treated db/db mice. **(D)** Serum insulin levels of mice from three groups after 5-week treatment of salidroside. **(E,F)** Effects of salidroside on the fasting glucose levels and body weights in HFD group mice. **(G)** After 5-week salidroside treatment, OGTT was performed in normal diet (ND), salidroside, or vehicle-treated HFD mice. **(H)** Serum insulin levels of mice from three groups after 5-week treatment of salidroside (^∗^*p* < 0.05, ^∗∗^*p* < 0.01 dbdb to dbdb/sal or HFD to HFD/sal). Data are shown as mean ± SE, *n* = 8.

To prevent progression of diabetes, we initially investigated effects of salidroside in 4-week db/db mice which are considered to be pre-diabetic. As showed in **Figure 1A**, salidroside could not significantly alleviate the increase of blood glucose in db/db mice in the first 15 days. However, after 21-day treatment, db/db mice were protected from developing severe hyperglycemia by salidroside and this effect was sustained in following days compared to vehicle db/db group. Moreover, there were no detectable changes in the body weights of salidroside treated db/db mice compared to vehicle db/db groups (**Figure 1B**). After the 5-week treatment, experiments of OGTT were performed. The response to OGTT was impaired in the db/db mice with a huge increase of blood glucose level (**Figure 1C**), and db/db mice treated with salidroside were significantly improved their tolerance to glucose compared to vehicle-treated db/db mice.

Another obese mouse model with metabolic syndrome, HFD mice were applied to test the function of salidroside as well, and 10-week HFD feeding induced a marked increase of fasting blood glucose in HFD mice compared with the normal diet (ND) mice (**Figure 1E**). Similarly, salidroside only showed hypoglycemic effect after 15 days treatment compared to the vehicles and its effect was enhanced with a longer treatment. Also, the body weight gain of HFD mice was unaffected by salidroside (**Figure 1F**). In addition, the improvement of OGTT in HFD mice with salidroside administration was observed (**Figure 1G**).

Meanwhile, an increasing tendency of fasting serum insulin levels was noticed in salidroside treated db/db or HFD mice, but no significant changes were found when compared with vehicle groups (**Figures 1D,H**).

### Salidroside Presented Antioxidant Activity in db/db and HFD Mice

**FIGURE 2:**
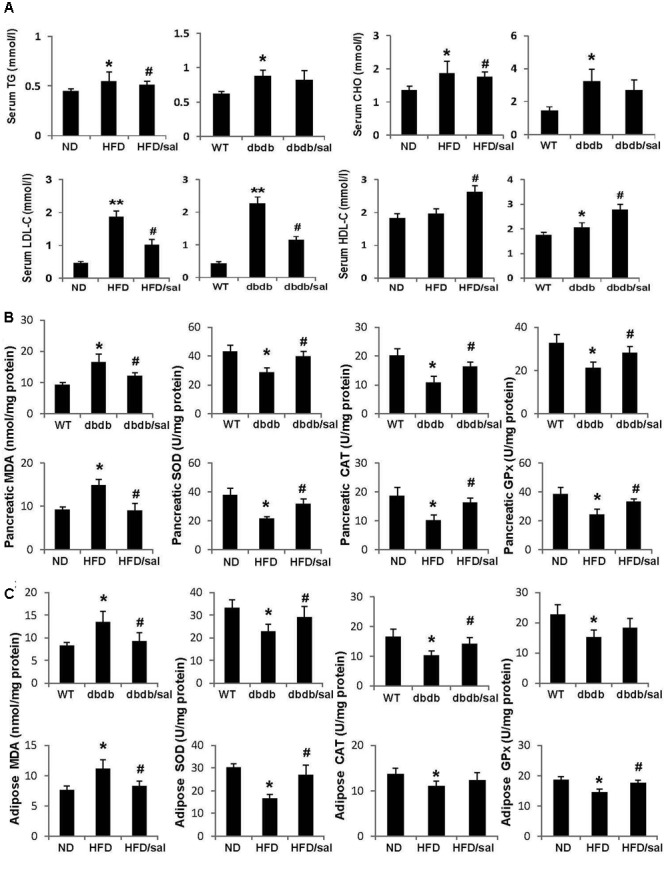
Salidroside presented anti-oxidant activities in db/db and HFD mice. **(A)** The serum triglyceride (TG), total cholesterol (CHO), low-density lipoprotein cholesterol (LDL-C), and high-density lipoprotein cholesterol (HDL-C) levels were examined in db/db and HFD mice. **(B)** Enzyme activities of SOD, CAT, GPx, and MDA content were measured in pancreas of mice. **(C)** Enzyme activities of SOD, CAT, GPx, and MDA content were measured in adipose tissue of mice. Data are shown as mean ± SE, *n* = 6 (^∗^*p* < 0.05, ^∗∗^*p* < 0.01, db/db to WT or HFD to ND, ^#^*p* < 0.05, dbdb to dbdb/sal or HFD to HFD/sal).

High-fat diet (HFD) and db/db mice are both obese animal models with elevated total CHO and TG contents. Here we noticed that salidroside slightly reduced total CHO or TG levels in HFD and db/db mice but without significant differences compared to vehicle groups (**Figure 2A**). However, salidroside significantly increased levels of HDL-C, but decreased levels of LDL-C in HFD and db/db mice.

A publication reported the protective effect of salidroside on oxidative stress in diabetic mice induced with a single injection of 200 mg/kg alloxan (Li et al., 2011). In this study, it was obtained that salidroside displayed strong antioxidant ability in pancreas of HFD and db/db mice as well. As presented in **Figure 2B**, a reduction in activity of antioxidant enzyme GPx, or SOD, or CAT was observed in pancreas of diabetic mice, while activities of these enzymes were enhanced by salidroside. On the other hand, MDA, a product of lipid peroxidation, was increased in diabetic mice, but was decreased by salidroside. Similar results were obtained in subcutaneous white adipose tissue of diabetic mice (**Figure 2C**). Salidroside significantly enhanced SOD activity and declined MDA content in adipose tissue of db/db and HFD mice.

### Salidroside Improved β-Cell Survival and Function under Diabetic Conditions

**FIGURE 3:**
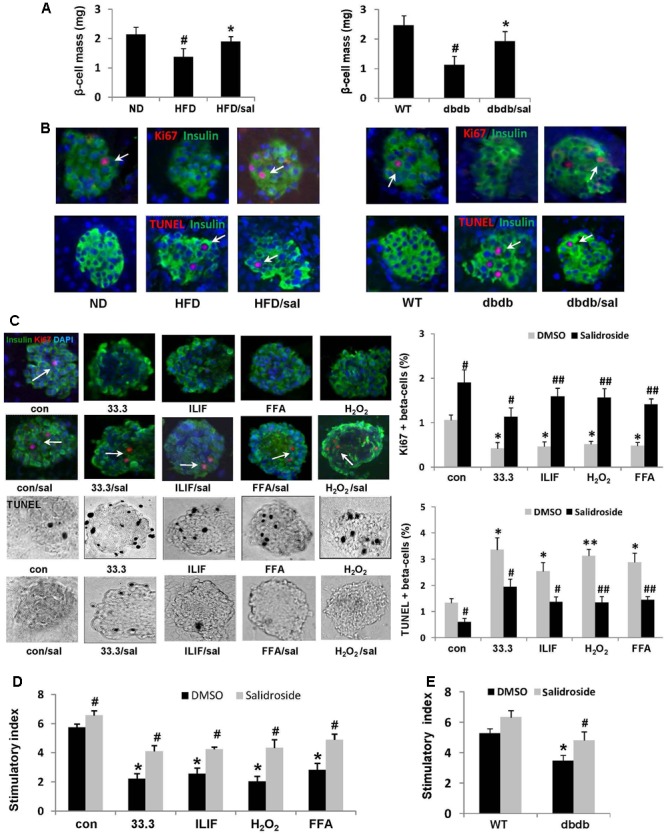
Salidroside improved β-cell survival under diabetic condition. **(A)** Five consecutive sections from each pancreas (6 mice per group) were used for β-cell mass measurements. Data are shown as mean ± SE, *n* = 6 (^#^*p* < 0.05 db/db to WT or HFD to ND, ^∗^*p* < 0.05 dbdb to dbdb/sal or HFD to HFD/sal). **(B)** Replication of β-cell in mice was examined in pancreatic sections by staining for Ki67 in red (indicated by white arrows), insulin in green, and DAPI in blue. β-cell apoptosis was detected by TUNEL staining in red (indicated by white arrows), insulin in green, and DAPI in blue. Representative pictures were shown. **(C)** Isolated mouse islets were exposed to diabetic stimuli [33.3 mM glucose or the mixture of 2 ng/ml IL-1β+1,000 U/ml IFN-γ (ILIF), or 0.5 mM palmitic acid], and H_2_O_2_ as a positive inducer of ROS (200 uM) with or without salidroside (50 μM) or DMSO as control for 3 days. Proliferation of β-cell was measured by the Ki67(red)/insulin(green)/DAPI(blue) co-staining (Ki67 positive staining was indicated by white arrows), and apoptosis by the TUNEL assay (black and white pictures, TUNEL positive presented as black dots). Results are expressed as means ± SE of the percentage of Ki67-positive or TUNEL-positive β-cells (^∗^*p* < 0.05, ^∗∗^*p* < 0.01 diabetic stimuli to control, **^#^***p* < 0.05, **^##^***p* < 0.01 salidroside to DMSO). **(D)** GSIS assay was performed in cultured mouse islets with different treatments. Stimulatory index denotes the amount of stimulated divided by the amount of basal insulin secretion. Data are shown as mean ± SE from three independent experiments (^∗^*p* < 0.05 diabetic stimuli to control, **^#^***p* < 0.05 salidroside to DMSO). **(E)** GSIS assay was performed in cultured mouse islets isolated from WT mice and db/db mice treated by salidroside or DMSO (^∗^*p* < 0.05 db/db to WT mice, **^#^***p* < 0.05 salidroside to DMSO).

As we reported before (Yang et al., 2016), here a significant reduction of β-cell mass in diabetic mice was found compared to ND or WT mice (**Figure 3A**). In contrast, an increase of β-cell mass was observed in salidroside treated group, which indicated the protective effect of salidroside on β-cell survival. Additionally, the positive staining of Ki67 in β-cells (a marker of cell proliferation) can be found in pancreas from diabetic mice treated by salidroside, but was hardly observed in mice treated by vehicle (**Figure 3B**). Meanwhile, β-cell apoptosis was examined by TUNEL staining (**Figure 3B**). TUNEL-positive β-cell was barely found in sections from both ND groups and WT group. The β-cell apoptosis was strongly increased in HFD and db/db mice, which was suppressed by salidroside. We postulate that β-cell survival improved by salidroside contributed to the increase of β-cell mass partially.

To further confirm the effect of salidroside on β-cell survival, we detected β-cell proliferation and apoptosis by Ki67/TUNEL co-staining using isolated mouse islets cultured with different diabetic stimuli. As shown in **Figure 3C**, high glucose (33.3), or the cytokines mixture of IL-1β/IFN-γ (ILIF), or palmitic acid (FFA, free fatty acid), or H_2_O_2_ (as a positive inducer of ROS) reduced β-cell proliferation, but induced β-cell apoptosis, compared with control group. On the contrary, islets can be protected by salidroside against these deleterious effects. Proliferation of β-cell was significantly increased by salidroside compared to DMSO-treated, and β-cell apoptosis was decreased simultaneously.

Moreover, glucose-stimulated insulin secretion (GSIS) assay revealed that the insulin secretion of cultured islets was strongly impaired by these different diabetic stimuli (**Figure 3D**). Salidroside, however, improved the function of islets by increasing the stimulatory index significantly. Likewise, the function of insulin secretion of islets isolated from db/db mice was enhanced by salidroside (**Figure 3E**). Our findings demonstrated salidroside was able to protect β-cell survival and function against diabetic stimuli.

### Salidroside Relieved Oxidative Stress to Improve β-Cell Survival by Decreasing NOX2 Expression and inactivating FOXO1

**FIGURE 4:**
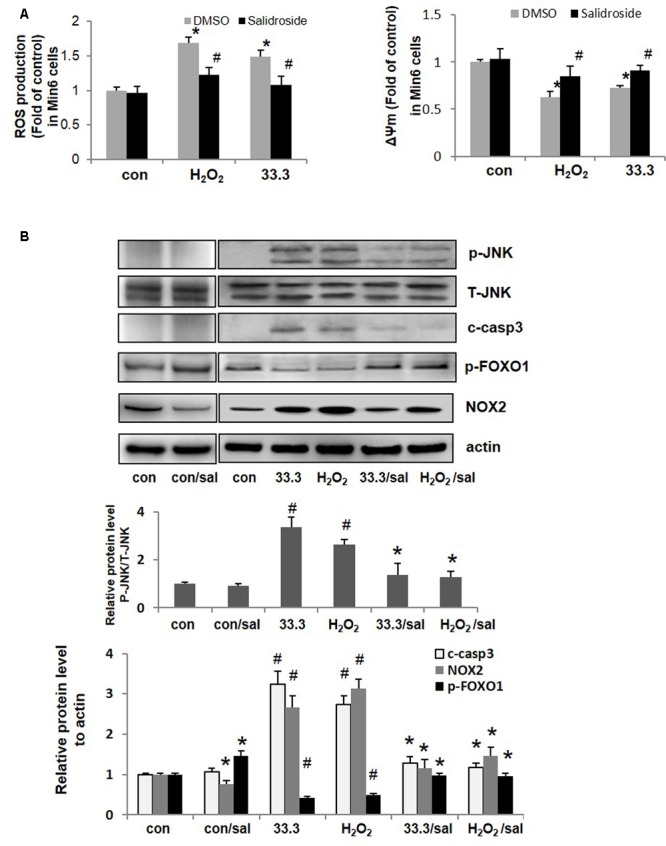
Salidroside relieved oxidative stress and inhibited apoptotic cascade in β-cell. **(A)** ROS production and mitochondrial membrane potential (ΔΨm) were detected in Min6 cells (^∗^*p* < 0.05 33.3 or H_2_O_2_ to control, **^#^***p* < 0.05 salidroside to DMSO). **(B)** Min6 cells were treated by 33.3 mM glucose or H_2_O_2_ (200 μM) with salidroside (50 μM) or DMSO for 3 days. Representative western blots from islets with different treatments were shown.

Direct exposure to oxidants such as H_2_O_2_ or secondary to glucolipotoxicity, inflammatory cytokines could lead to oxidative stress in β-cell. Here increased ROS production was detected in Min6 cells treated by high glucose or H_2_O_2_, the destroyed ΔΨm (mitochondrial membrane potential) was found in Min6 cells as well. As a natural antioxidant, salidroside effectively suppressed ROS production and recovered ΔΨm in β-cells compared to DMSO-treated cells (**Figure 4A**).

NADPH oxidase (NOX) is a key regulator of ROS production and mediates ROS triggered cell apoptosis. As presented in **Figure 4B**, NOX2 was up-regulated by high glucose or H_2_O_2_ in Min6 cells, followed by increased levels of p-JNK and cleaved caspase 3 which resulted in β-cell apoptosis finally. However, salidroside down-regulated NOX2 expression and inhibited subsequent activations of JNK and caspase 3 to prevent β-cell death.

Studies have shown that inactivation of FOXO1 contributes to β-cell protection under oxidative damage (Ding et al., 2014; Shao et al., 2014). It is known that in β-cells FOXO1 acts as a negative regulator of PDX1 functions and mediate β-cell dysfunction and apoptosis. Phosphorylation of FOXO1 (Ser256) indicated the function loss of it. Interesting, here we noticed that the p-FOXO1 (Ser256) level was up-regulated by salidroside (**Figure 4B**). Next the impacts of salidroside on FOXO1/PDX1 were investigated.

### Salidroside Protected β-Cells via AMPK Mediated Inhibitions of FOXO1 and NOX2

**FIGURE 5:**
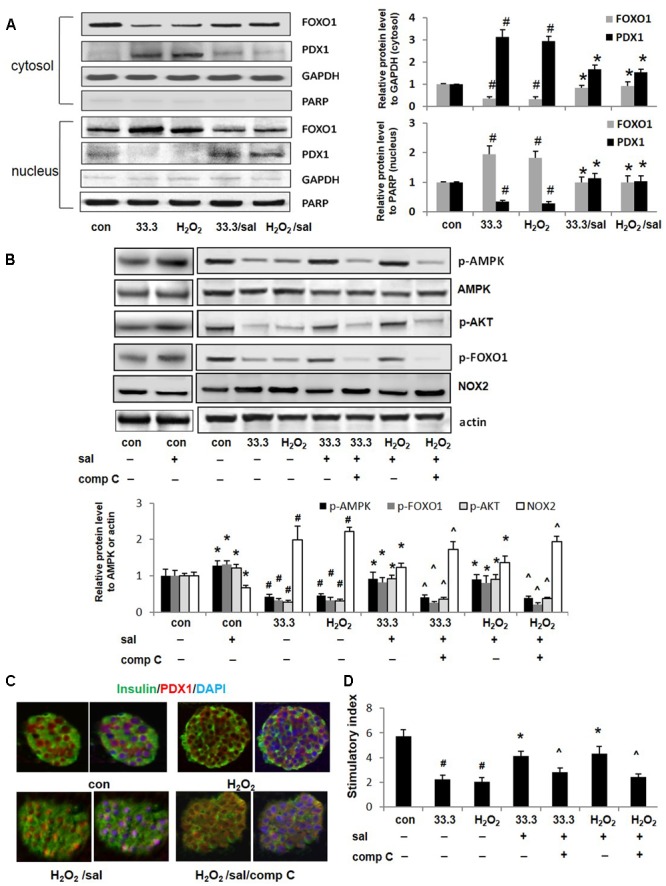
Salidroside improved PDX1 nuclear localization and β-cell function depended on AMPK activation. **(A)** The cellular distributions of FOXO1 and PDX1 were detected by western blotting analysis in Min6 cells treated by high glucose or H_2_O_2_ for 2 days. **(B)** Effects of compound C (10 μM) on phosphorylations of AMPK, AKT, FOXO1, and expression of NOX2 in Min6 cells were examined by western blot analysis. The densitometry analyses of three independent experiments are shown. **(C)** Isolated mouse islets were exposed to H_2_O_2_ with salidroside or compound C (comp C), DMSO as control for 12 h. Islets were triple-stained for PDX1 in red, insulin in green, and DAPI in blue to detect the PDX1 localization. **(D)** GSIS assay was performed in treated mouse islets with/without salidroside. Stimulatory index denotes the amount of stimulated divided by the amount of basal insulin secretion. Data are shown as mean ± SE from three independent experiments (**^#^***p* < 0.05 33.3 or H_2_O_2_ to control, ^∗^*p* < 0.05 salidroside to DMSO, and ˆ*p* < 0.05 Sal/comp C to Sal).

The PDX1 is an essential transcription factor for maintaining β-cell function and survival. Impairment of PDX1 nuclear localization results in β-cell dysfunction. Here we found in Min6 cells treated by high glucose or H_2_O_2_, PDX1 predominantly accumulated in the cytoplasm fraction, while most of FOXO1 proteins located in the nucleus (**Figure 5A**). However, with salidroside treatment, PDX1 translocated into the nucleus, and nuclear exclusion of FOXO1 was observed. The nuclear/cytosolic ratio of PDX1 was significantly elevated by salidroside compared to DMSO-treated cells.

The AKT could suppress the function of FOXO1 by directly phosphorylating it at Ser256. As expected, here we observed that salidroside activated AKT by increasing p-AKT level (**Figure 5B**). More importantly, we noticed that FOXO1 phosphorylation induced by salidroside was correlated with AMPK activation. Compound C, an inhibitor of AMPK, blocked up-regulations of p-AMPK and p-AKT stimulated by salidroside. The FOXO1 phosphorylation was suppressed by compound C simultaneously. Interesting, here we found the decrease of NOX2 expression induced by salidroside was correlated to AMPK activation and was suppressed by compound C as well (**Figure 5B**). Quercetin, a bioactive natural compound, was reported could decrease NOX2 expression through activating AMPK as a potent antioxidant (Hung et al., 2015). Our findings provide another evidence to support the association between AMPK and NOX2.

Further investigations confirmed that PDX1 nuclear localization promoted by salidroside was AMPK activation dependent, which could be blocked by compound C (**Figure 5C**). Additionally, β-cell function of insulin secretion improved by salidroside was diminished by compound C as well (**Figure 5D**).

Given these findings, we assume that salidroside may protect β-cell survival and function through AMPK mediated inhibitions of NOX2 and FOXO1 simultaneously.

## Discussion

Growing evidence has shown robust oxidative stress, caused either by direct exposure to oxidants such as H_2_O_2_ or secondary to glucolipotoxicity, leads to β-cell dysfunction and β-cell death ultimately. Chronic oxidative stress is a mediator for glucose toxicity of the β-cell in T2DM (Robertson et al., 2007). Elevated plasma FFA levels that are presented in obesity also result in increased ROS generation in β-cells (Koshkin et al., 2003). In T2DM Zucker diabetic fatty rats, increased ROS generation is associated with the onset of diabetes (Syed et al., 2011). The β-cell has very low intrinsic levels of antioxidant proteins with low enzyme activity, thus β-cell is very vulnerable to ROS (Robertson et al., 2007). Treatment with antioxidants could protect animal models of T2DM against development of phenotypic hyperglycemia (Tanaka et al., 1999).

Medicinal plants and natural compounds are important resources for healthcare and treatments of diabetes (Xiao, 2015; Chen et al., 2017). As a natural antioxidant, the effects of salidroside on improving insulin resistance and suppressing hepatic glucose production have been investigated very well (Li et al., 2008; Lee et al., 2015; Zheng et al., 2015). However, the direct effect of salidroside on β-cells and the underlying mechanisms are poorly understood.

**FIGURE 6:**
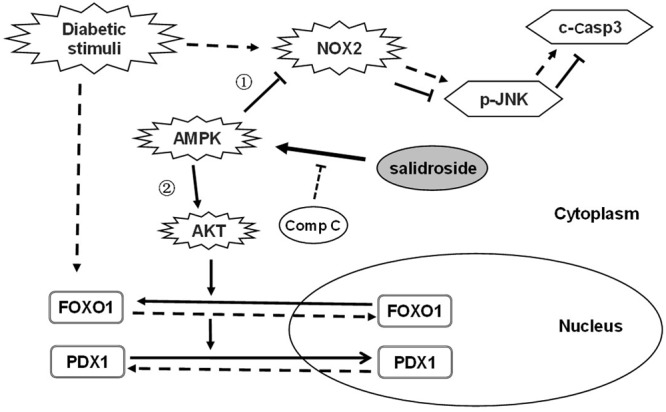
Proposed action pathway of salidroside in preventing β-cell failure under diabetic oxidative stress. Straight line arrow (→) represent actions of salidroside and dotted line arrow (--→) represent actions of diabetic stimuli.

In this study, for the first time, we demonstrate protective effects of salidroside on β-cell survival and function against diabetic stimuli. A proposed action pathway of salidroside was shown in **Figure 6**: (1) Salidroside protected β-cell survival by activating AMPK to reduce NOX2 expression and suppress activation of JNK–caspase 3 apoptotic cascade subsequently. (2) Salidroside activates AMPK-AKT to inhibit FOXO1 which recovers PDX1 function and prevent β-cell dysfunction. The role of AMPK in β-cell function is controversial (Fu et al., 2013). Our study explored the function of AMPK on β-cell function and survival to extend the understanding of the role of AMPK in β-cells.

The NADPH oxidases have been reported to be responsible for increased ROS generation in the T2DM Zucker diabetic fatty rat (Syed et al., 2011). NOX2 is the predominant isoform to modulate ROS generation in β-cells (Li et al., 2012). In cultured mouse islets, we observed NOX2 expression was up-regulated by high glucose or H_2_O_2_, while salidroside inhibited the increase of NOX2, suppressed activations of JNK and caspase 3 subsequently, and prevented β-cell apoptosis eventually.

The relationship between AMPK and NOX2 has been reported by many publications. For example, it was shown that AMPK activation could prevent NOX2 activation induced by hyperglycemia in adult cardiomyocytes (Balteau et al., 2014). Another study reported that AMPK activation protected against homocysteine-induced apoptosis of osteocytic MLO-Y4 cells by regulating expressions of NOX1 and NOX2 (Takeno et al., 2015). Quercetin, a bioactive natural compound, was reported and could decrease NOX2 expression through activating AMPK as a potent antioxidant (Hung et al., 2015). The present study provided another evidence to support the association between AMPK and NOX2.

The PDX1 is a critical transcription factor that positive-regulates β-cell survival and function (Sachdeva et al., 2009). Impaired PDX1 location in the nucleus has been suggested as a mechanism of β-cell failure in diabetes. FOXO1 and PDX1 exhibit mutually exclusive patterns of nuclear localization in β-cells, and constitutive nuclear expression of a mutant FOXO1 is associated with lack of PDX1 expression (Kitamura et al., 2002). Being similar to a previous report (Kawamori et al., 2003), in this study, PDX1 shuttling from the nucleus to the cytosol was induced by high glucose or H_2_O_2_, and nuclear accumulation of FOXO1 was observed. In contrast, salidroside remarkably prevented nuclear exclusion of PDX1, thus recovered the function of PDX1 to improve the β-cell function.

In recent years, the role of AMPK in regulating gluconeogenesis and insulin resistance in T2DM has been increasingly appreciated. For example, metformin acts by stimulating AMPK, leading to reduced glucose production in the liver and reduced insulin resistance in the muscle (Zhou et al., 2001). It has been shown salidroside ameliorates hepatic glucose production and insulin resistance through activation of AMPK/AKT signaling (Li et al., 2008; Zheng et al., 2015). We confirmed salidroside increased levels of p-AMPKα and p-AKT in β-cell. Interestingly, here we noticed that in Min6 cells, the inactivation of FOXO1 caused by salidroside was related to activation of AMPK/AKT. Increased p-AMPKα (Thr172) and p-FOXO1 (Ser256) were also found in a study of low-branched-chain amino acids regulating hepatic fatty acid metabolism (Bai et al., 2015). In addition, compound C, an inhibitor of AMPK, could block the increase of p-AMPK or p-FOXO1 induced by salidroside. The improvements of salidroside on PDX1 and β-cell function were abolished by compound C as well. Compound C has been wildly used as a specific inhibitor of AMPK in many research fields. However, a study reported compound C reduced glioma viability in an AMPK-independent way (Liu et al., 2014). To confirm the critical role of AMPK in effects of salidroside, AMPK expression knock-down by siRNA transfection will be applied in our future study.

FOXO1 is constitutively active in diabetic mice, leading to increased hepatic glucose production and fasting hyperglycemia (Altomonte et al., 2003). The beneficial effect of FOXO1 inhibition in db/db mice on glucose homeostasis is being recognized (Nagashima et al., 2010). Similar report showed that curcumin protected Min6 β-cells against apoptosis induced by palmitate through inhibition of nuclear translocation of FOXO1 (Hao et al., 2015). Our study provided another evidence to support the hypothesis of developing FOXO1 as a new target for diabetic therapy.

## Conclusion

Our present findings clarify a novel role of salidroside in preventing β-cell failure via AMPK activation. Our finding highlights the potential value of salidroside to maintain β-cell mass in type 2 diabetes. However, the detailed molecular mechanism of how salidroside regulates AMPK activation still needs to be elucidated in our future study.

## Author Contributions

LJ, XW, CW, YD, YP, and LS: Performed experiments and analyzed the data. YW and LF: Contributed the analytic tools. LS: Designed experiments and wrote the paper.

## Conflict of Interest Statement

The authors declare that the research was conducted in the absence of any commercial or financial relationships that could be construed as a potential conflict of interest.
